# Effects of Implementing a Barcode Information Management System on Operating Room Staff: Comparative Study

**DOI:** 10.2196/56192

**Published:** 2024-10-17

**Authors:** Chia-Yen Li, Mei-Hui Huang, Yu-Shiue Lin, Chi-Ming Chu, Hsueh-Hsing Pan

**Affiliations:** 1 Graduate Institute of Medical Sciences National Defense Medical Center Taipei Taiwan; 2 Department of Planning and Management Tri-Service General Hospital Taipei Taiwan; 3 Department of Nursing Tri-Service General Hospital Taipei Taiwan; 4 Department of Anesthesia Tri-Service General Hospital Taipei Taiwan; 5 Graduate Institute of Life Sciences, School of Public Health National Defense Medical Center Taipei Taiwan; 6 Big Data Research Center College of Medicine Fu-Jen Catholic University New Taipei City Taiwan; 7 Department of Public Health China Medical University Taichung Taiwan; 8 Department of Healthcare, Department of Healthcare Administration and Medical Informatics College of Health Sciences Kaohsiung Medical University Kaohsiung Taiwan; 9 Department of Medical Research Kaohsiung Medical University Hospital Kaohsiung Taiwan; 10 School of Nursing National Defense Medical Center Taipei Taiwan

**Keywords:** barcode information management system, barcode, information system, operation management information system, Agile development model

## Abstract

**Background:**

Barcode information management systems (BIMS) have been implemented in operating rooms to improve the quality of medical care and administrative efficiency. Previous research has demonstrated that the Agile development model is extensively used in the development and management of information systems. However, the effect of information systems on staff acceptance has not been examined within the context of clinical medical information management systems.

**Objective:**

This study aimed to explore the effects and acceptance of implementing a BIMS in comparison to the original information system (OIS) among operating and supply room staff.

**Methods:**

This study was a comparative cohort design. A total of 80 staff members from the operating and supply rooms of a Northern Taiwan medical center were recruited. Data collection, conducted from January 2020 to August 2020 using a mobile-based structured questionnaire, included participant characteristics and the Information Management System Scale. SPSS (version 20.0, IBM Corp) for Windows (Microsoft Corporation) was used for data analysis. Descriptive statistics included mean, SD, frequency, and percentage. Differences between groups were analyzed using the Mann-Whitney *U* test and Kruskal-Wallis test, with a *P* value <.05 considered statistically significant.

**Results:**

The results indicated that the BIMS generally achieved higher scores in key elements of system success, system quality, information quality, perceived system use, perceived ease of use, perceived usefulness, and overall quality score; none of these differences were statistically significant (*P*>.05), with the system quality subscale being closest to significance (*P*=.06). Nurses showed significantly better perceived system use than technicians (1.58, SD 4.78 vs –1.19, SD 6.24; *P*=.02). Significant differences in perceived usefulness were found based on educational level (*P*=.04) and experience with OIS (*P*=.03), with junior college-educated nurses and those with over 6 years of OIS experience reporting the highest perceived usefulness.

**Conclusions:**

The study demonstrates that using the Agile development model for BIMS is advantageous for clinical environments. The high acceptance among operating room staff underscores its practicality and broader adoption potential. It advocates for continued exploration of technology-driven solutions to enhance health care delivery and optimize clinical workflows.

## Introduction

The implementation of barcode information management systems (BIMS) in clinical practice is widely recognized as a method to enhance health care quality and safety [1]. BIMS replaces manual processes, thereby improving efficiency, quality management, and reducing costs [2]. They are used for patient identification, medication administration, and specimen transportation, contributing to reduced nursing turnover and increased patient satisfaction [3-5]. As health care quality becomes more important, organizations are upgrading management practices.

Despite the initial costs and potential staff resistance, technologies such as barcodes have demonstrated significant improvements in hospital services [6]. Health information technology (HIT) is significant for effectively managing and improving medical care quality, reducing costs, and addressing various challenges. HIT encompasses a range of technologies used to manage health information, including electronic health records, clinical decision support systems, and BIMS [7,8]. Effective HIT systems ensure that different technologies can communicate and share data seamlessly, which is critical for coordinated and efficient care [9,10]. However, challenges associated with HIT include cost, interoperability, privacy and security, data quality, and workflow integration [7,8].

The advantages of HIT are well-documented, including the ease of reading and interpreting physiological values; reduced time for completing electronic records; and decreased errors in drug administration, blood drawing, and specimen transportation. Consequently, HIT can improve patient safety, enhance nursing efficiency, and increase both nurse and patient satisfaction [3,4,11]. A previous study has shown that barcode systems can reduce surgical instrument packaging time and costs [12], and further research has demonstrated that HIT advances the safety and quality of surgical care, reduces staff stress, and enhances service quality [3,13].

Operating rooms are high-cost, high-skill units where complex and critical issues can significantly impact patient safety. It is essential for operating room nurses to maintain a positive view of patient care, ensure the physical safety of patients, and consider patient vulnerabilities [14]. The quality and efficiency of services in operating rooms are of paramount concern, as surgeries are invasive procedures that require stringent sterilization of medical equipment to prevent nosocomial infections. Such infections can seriously compromise patient safety, prolong hospitalization, and increase medical costs, thereby affecting a hospital’s reputation [11]. The National Healthcare Safety Network [15] reported that surgical site infections are the costliest health care–associated infections, accounting for 20% of such infections and increasing the risk of death significantly. Improving surgical safety is one of Taiwan’s patient safety goals, aimed at enhancing high-quality medical care and avoiding unnecessary patient harm [16].

In the software industry, Agile methods are widely adopted for efficient product and service development [17]. Agile software development (ASD) has become the primary approach for managing information systems implementation, which is crucial for modern digital health software. ASD facilitates customer feedback and revisions, emphasizing teamwork and collective decision-making in cross-functional teams [18,19]. The Agile transition process can be modified to adapt to the original standard process but may face challenges primarily due to human factors [19,20]. Issues such as a lack of understanding of the development model, direct implementation without comprehension, adherence to familiar practices, and disagreement with development values can lead to resistance and a failure to consider the entire process [19].

The relationship between BIMS, HIT, and ASD is interconnected, with each component playing a crucial role in modernizing and optimizing health care delivery. BIMS, as a part of HIT, relies on the robust infrastructure provided by HIT systems to ensure seamless integration and effective data use [21,22]. ASD supports the development and refinement of BIMS by emphasizing flexibility, user feedback, and iterative improvements, ensuring that BIMS solutions are continuously optimized based on real-world usage and feedback from health care staff [23]. Effective assessment is crucial when using ASD in operational engineering, as it helps identify team issues and facilitates improvements to enhance user experience. In the context of surgery, information systems manage logistics and materials, involving various professionals such as nurses and technicians. Therefore, this study explores the effects of implementing ASD with BIMS on operating room staff, providing insights into operational management and the comparative impact on staff experiences and efficiencies.

## Methods

### Study Design, Setting, and Participants

This study uses a comparative cohort design to evaluate the effects of implementing a BIMS on operating room staff, including nurses and technicians. The study was conducted between January 2020 and October 2020.

Participants were recruited from the operating and supply rooms of a medical center in Northern Taiwan. A total of 126 staff members, including nurses and technicians responsible for handling sterile device materials or packaging and checking expiration dates, were eligible to participate. Therefore, a census approach was used for this group. Ultimately, 80 staff members, comprising 48 nurses and 32 technicians, agreed to participate and were included in the analysis.

### Operation Management Information System

In surgical procedures, various clinical personnel collaborate to ensure seamless operations. The supply room, a vital medical logistics unit, manages the cleaning, packaging, sterilization, storage, and supply of medical devices, including expiration date management. Staffed by nurses and technicians, this unit ensures adherence to infection control principles for patient safety and efficient surgical procedures. The introductions of the original information system (OIS) and BIMS was as follows.

### Original Information System

Under the OIS, the outpatient and emergency information systems are not connected to the surgical information system, requiring doctors to manually enter the surgical schedule repeatedly. The day before surgery, an operating room nurse selects the surgery case cart, specialized equipment, and packaging based on the surgery requirements. When additional packages, specialized tools, equipment, or materials are needed during surgery, nurses must select items and quantities in the original system or generate a handwritten list. They then confirm the material dynamics of temporary selections with the supply room by phone. The lack of immediate access to packaging sterilization information and inventory levels necessitates the estimation of item locations and sterilization processes through paper records, resulting in significant time spent tracking for the supply room.

### Barcode Information Management System

The BIMS significantly enhances these operations. A doctor imports the surgery schedule and selects the specialist package and unique device identification system final rule. The day before surgery, an operating room nurse confirms the patient’s case cart, special package, and required materials. During surgery, if additional packages or specialized tools are needed, nurses use BIMS to select the necessary items and quantities. Technicians then receive urgent messages from BIMS, acquire the supplies, and deliver them to the operating room.

BIMS streamlines communication and tracking, allowing nurses to monitor material processing status conveniently without needing to write application forms or confirm over the phone. It provides detailed information about sterilization equipment and the usage history of sterilized packages, enabling instant tracking of item locations and sterilization processes, along with managing package validity periods. This eliminates the need for phone confirmation with the supply room. If a system warning indicates insufficient supply allocation and the technician cannot resolve it directly, it is reported back to nurses. They use the system to understand device status dynamics and coordinate the process with the clinician.

**Figure 1:**
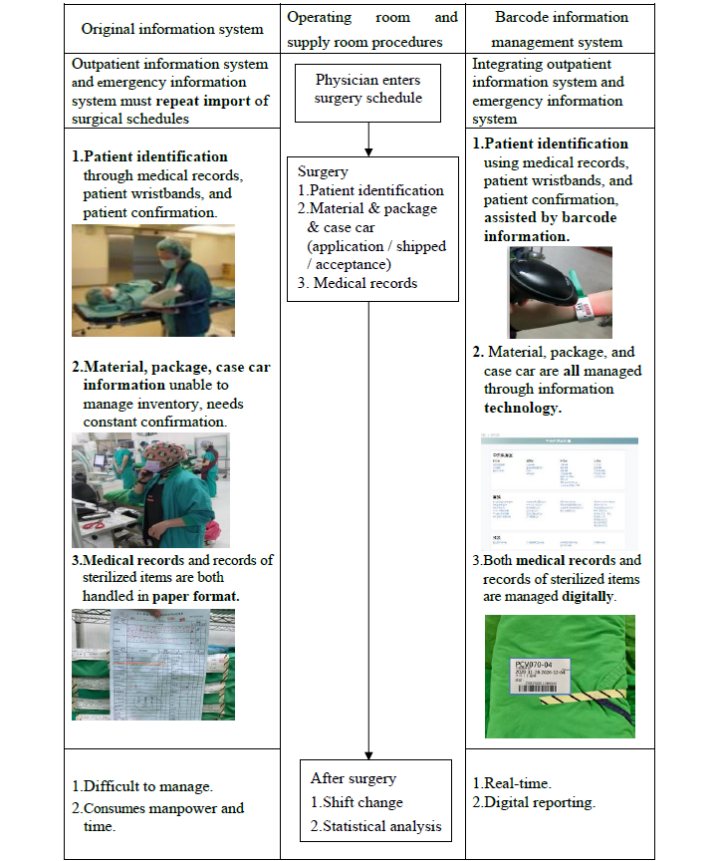
Comparison between the original information system and barcode information management system.

Nurses oversee sterilization quality control, communicate with clinical units, and guide technical staff. They coordinate staff, manage instruments, handle supply alerts, assist in surgeries, provide patient care, and ensure the sterilization of equipment. Technicians are responsible for cleaning, packaging, sterilizing, and supplying instruments. This collaboration, facilitated by BIMS, is crucial for maintaining efficient and safe surgical procedures. Figure 1 describes the comparison between OIS and BIMS in the surgical procedure.

### Measurements

#### Characteristics of Participants

The characteristics of the participants included the following variables: identity, sex, and age (categorized into 3 groups: young [19-34 years], early middle-aged [35-49 years], and late middle-aged adults [50-65 years]) [24], educational level (below senior high school, specialist, and junior college), working years, and experience using the original system.

#### Information Management System Scale

The Information Management System Scale (IMSS) developed by Chiang and Lee [25] was used to evaluate the effectiveness of the nursing staff information system. The IMSS comprises 28 questions divided into 3 main parts.

The “key elements of system success” section includes 2 subscales: system quality, with 7 questions focusing on screen simplicity, data classification, reading speed, response time, screen transition, function availability, and patient care information integration; and information quality, with 7 questions on team communication efficiency, time savings, information accuracy, and display quality.

The “perceived system use” section also has 2 subscales: perceived ease of use, with 5 questions on system operation, use, proficiency attainment, and the system’s significance in improving clinical work efficiency and team connectivity; and perceived usefulness, with 5 questions on the system’s convenience in clinical work, control of patient conditions, work quality improvement, procedure simplification, and usefulness of patient information.

The “attitude toward system use” section includes 4 questions assessing the information system as a valuable tool for the medical team and its role in improving care quality.

A Likert scale ranging from 1 (strongly disagree) to 5 (strongly agree) was used, with higher scores indicating greater satisfaction, efficiency improvement, and positive attitudes. The scale was tested with 185 nurses, achieving an overall Cronbach α of 0.973, with subscale Cronbach α values between 0.897 and 0.920 [25]. This study invited an information supervisor, an information engineer, and a senior nurse to validate the assessments on a scale of 1 to 5 for correctness, suitability, and completeness of the questionnaire contents, resulting in a content validity index of 0.94. We recruited 80 participants for this study, achieving an overall Cronbach α of 0.984, with each subscale having a Cronbach α ranging from 0.925 to 0.969, indicating high reliability and validity.

### Statistical Analysis

The SPSS (version 20; IBM Corp) software for Windows (Microsoft Corporation) was used to analyze the data. Continuous variables were presented as mean and SD, while categorical variables were presented as frequencies and percentages. The Mann-Whitney *U* test and Kruskal-Wallis test were used for univariate analysis to compare differences in participants’ data. A *P* value of <.05 was considered statistically significant.

### Ethical Considerations

**Figure 2:**
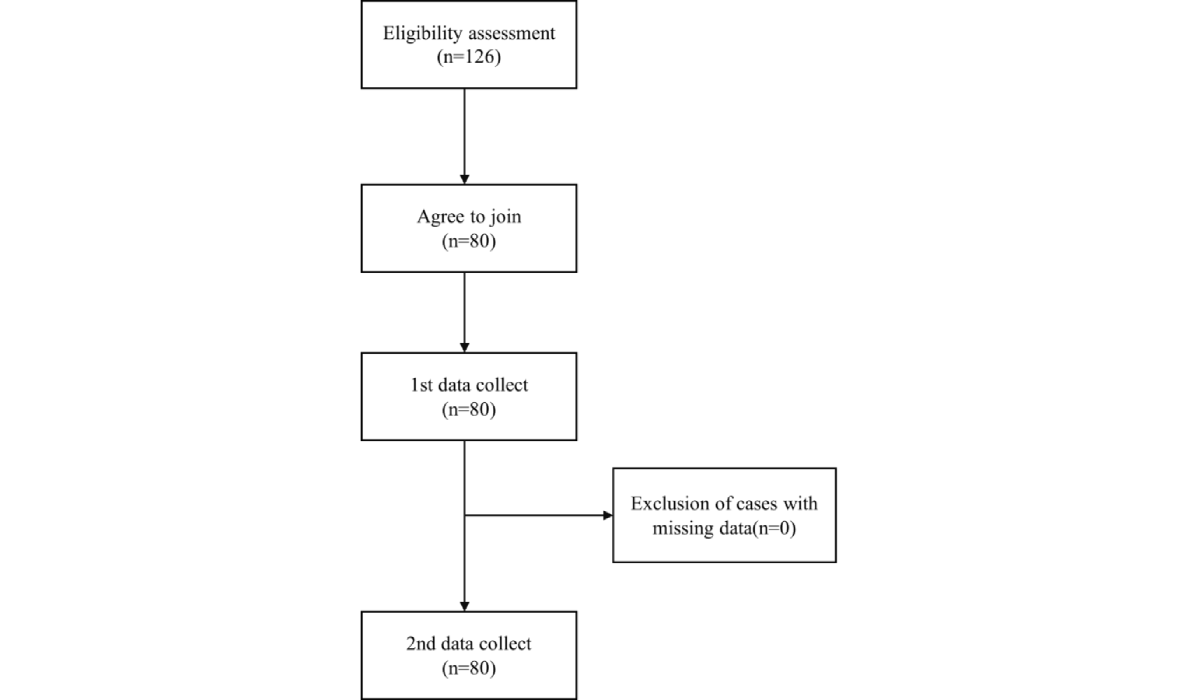
Flowchart of the study population.

This study was approved by the Institutional Review Board of Tri-Services General Hospital (No. C202005035). The purpose of the study was explained by the same researcher to the operating room and supply room staff of a medical center in northern Taiwan during morning meetings. Out of 126 eligible participants, 80 agreed and signed the informed consent to participate in the study. Data was collected both before (using OIS) and after the implementation of the BIMS. Baseline data was collected in January 2020, and post-implementation data were collected in August 2020. This approach allowed for a comprehensive comparison of the effects of BIMS on the operating and supply room staff, including nurses and technicians. The details are shown in Figure 2.

## Results

### Characteristics of Participants

A total of 80 staff members from the operating and supply rooms participated in the study, comprising 42 nurses (60%) and 38 technicians (40%). The mean age of the participants was 47.1 (SD 10.4) years. Most participants were female (n=64, 80%) and had a bachelor’s degree or above (n=34, 42%). In addition, 46% had over 20 years of service experience, and 71% (n=57) had more than 6 years of experience using the OIS. Table 1 presents these details.

**Table 1:** Characteristics of participants (N=80).

Variables		Participants
**Identity, n (%)**		
	Nurse	48 (60)
	Technician	32 (40)
**Sex** **n (%)**		
	Male	16 (20)
	Female	64 (80)
**Age (years), mean (SD)**		47.1 (10.4)
	Young adults (19-34), n (%)	8 (10)
	Early middle-aged adults (35-49), n (%)	39 (49)
	Late middle-aged adults (50-65), n (%)	33 (41)
**Educational level, n (%)**		
	Below senior high school	24 (30)
	Junior college	22 (28)
	Bachelor and above	34 (42)
**Length of service year (years), mean (SD)**		18.3 (12)
	<10, n (%)	25 (31)
	10-20, n (%)	18 (23)
	>20, n (%)	37 (46)
**Experience of using original information systems (years), n (%)**		
	<3	19 (24)
	3-6	4 (5)
	>6	57 (71)

### Difference Between Original Information System and Barcode Information Management Systems in Information Management System Scale Scores

The results, as presented in Table 2, show that the BIMS generally achieved higher scores compared with the OIS across various metrics. While BIMS showed improvements in key elements of system success, system quality, information quality, perceived system use, perceived ease of use, perceived usefulness, and overall quality score, none of these differences were statistically significant (*P*>.05). The closest to significance was the system quality subscale (*P*=.06). Attitudes toward system use were similarly positive for both systems. Overall, BIMS demonstrated a trend toward better performance, but without significant statistical differences from OIS.

**Table 2:** Comparison of original information system and barcode information management systems Information Management System Scale scores (N=80).

Variable (item)			Original information system					Barcode information management systems					*z*			*P* value
			Total, mean (SD)		Average, mean (SD)		Total, mean (SD)			Average, mean (SD)						
**Informational Management System Scale**																
	Key elements of system success	48.51 (9.96)		3.46 (0.71)		49.88 (8.55)			3.56 (0.61)		–1.38			.17		
	System quality (7)	24.13 (4.78)		3.45 (0.68)		25.01 (4.11)			3.57 (0.58)		–1.92			.06		
	Information quality (7)	24.37 (5.38)		3.48 (0.77)		24.87 (4.57)			3.55 (0.65)		–0.81			.42		
	Perceived system use	35.46 (7.11)		3.54 (0.71)		35.94 (5.76)			3.59 (0.57)		–0.79			.43		
	Perceived ease of use (5)	17.71 (3.69)		3.54 (0.73)		17.93 (3.04)			3.58 (0.60)		–0.58			.56		
	Perceived usefulness (5)	17.75 (3.63)		3.46 (0.71)		18.02 (2.91)			3.60 (0.58)		–0.81			.42		
	Attitudes of system use (4)	18.25 (3.74)		4.56 (0.94)		17.96 (3.42)			4.49 (0.86)		–0.58			.56		
	Overall quality score	102.23 (19.83)		3.65 (0.71)		103.78 (17.15)			3.71 (0.61)		–0.85			.39		

### Difference Between Original Information System and Barcode Information Management Systems According to Participant Characteristics, Key Elements of System Success, Perceived System Use, and Attitude Toward System Use

The difference between the OIS and BIMS was calculated by deducting the OIS score from the BIMS score across various participant characteristics and key elements of system success, perceived system use, and attitudes toward system use among 80 participants. A positive difference indicates a better information management system using BIMS. Significant differences were observed in participant identity and perceived system use, with nurses showing better perceived system use than technicians (1.58, SD 4.78 vs –1.19, SD 6.24; *P*=.02). No statistically significant differences were observed in gender, age, educational level, length of service, or experience in using an OIS (*P*>.05). Table 3 lists these details.

Table 4 shows the differences between the BIMS and OIS scores according to participant characteristics and the 2 subscales, perceived ease of use and perceived usefulness, of the perceived system use measure, were also investigated. The results revealed significant differences between the participant’s identity and perceived usefulness. Nurses had better perceived the system’s usefulness than technicians (0.85, SD 2.46 vs –0.63, SD 3.39; *P*=.03). No statistically significant differences were observed in the demographics, perceived ease of use, and perceived usefulness (*P*>.05).

Table 5 lists the differences in participant characteristics and perceived usefulness between nurses and technicians using BIMS and OIS. The results showed significant differences in perceived usefulness based on educational level (*P*=.04) and experience in using the OIS (*P*=.03). Junior college–educated nurses reported the highest perceived usefulness. In addition, nurses with more than 6 years of experience using the OIS reported higher perceived usefulness compared with technicians.

**Table 3:** Comparison of original information system and barcode information management systems scores for participant characteristics and key elements of system success, perceived system use, and attitude toward system use (N=80).

Variable		Key elements of system success^a^				Perceived system use^a^				Attitudes of system use^a^		
		Mean (SD)	*P* values		Mean (SD)		*P* values		Mean (SD)		*P* values	
**Identity**				.14^b^				.02^b^				.19^b^
	Nurse	2.27 (7.04)			1.58 (4.78)				0.12 (2.92)			
	Technician	0.03 (9.98)			-1.19 (6.24)				-0.91 (4.11)			
**Sex**				.47^b^				.53^b^				.26^b^
	Male	–0.19 (8.26)			–1.00 (6.25)				–1.31 (4.09)			
	Female	1.77 (8.39)			0.84 (5.34)				–0.03 (3.27)			
**Age**				.71^c^				.30^c^				.90^c^
	Young adults	2.75 (68.6)			1.75 (4.13)				0.13 (2.17)			
	Early middle-aged adults	1.69 (7.30)			0.89 (5.56)				–0.62 (3.64)			
	Late middle-aged adults	0.67 (9.87)			–0.33 (5.85)				0.00 (3.54)			
**Educational level**				.83^c^				.24^c^				.27^c^
	Below senior high school	1.29 (10.88)			–0.75 (6.64)				–0.71 (4.53)			
	Junior college	2.23 (6.99)			1.82 (4.56)				0.50 (2.81)			
	Bachelor and above	0.88 (7.26)			0.47 (5.24)				–0.50 (2.97)			
**Length of service years**				.55^c^				.24^c^				.18^c^
	<10 years	–0.16 (6.99)			–1.12 (5.53)				–1.36 (3.43)			
	10-20 years	1.44 (8.35)			1.59 (5.62)				–0.26 (3.32)			
	>20 years	2.68 (9.46)			0.82 (5.36)				0.64 (3.46)			
**Experience of using original information system**				.43^c^				.11^c^				.16^c^
	<3 years	–0.58 (7.51)			–1.63 (5.91)				–1.68 (3.59)			
	3-6 years	–1.00 (2.94)			–1.50 (2.38)				–1.25 (2.62)			
	>6 years	2.19 (8.80)			1.32 (5.42)				0.24 (3.37)			

^a^Difference between original information system and barcode information management system.

^b^Mann-Whitney *U* test.

^c^Kruskal-Wallis test.

**Table 4:** Comparison of original information system and barcode information management system scores for participant characteristics and perceived system use, including perceived ease of use and perceived usefulness (N=80).

Variables		Perceived system use^a^			
		Perceived ease of use		Perceived usefulness	
		Mean (SD)	*P* values	Mean (SD)	*P* values
**Identity**			.09^b^		.03^b^
	Nurse	0.73 (2.84)		0.85 (2.46)	
	Technician	–0.56 (3.32)		–0.63 (3.39)	
**Sex**			.99^b^		.45^b^
	Male	–0.18 (3.51)		–0.81 (3.27)	
	Female	0.31 (2.99)		0.53 (2.82)	
**Age (years)**			.34^c^		.41^c^
	Young adults	0.87 (2.99)		0.87 (1.45)	
	Early middle-aged adults	0.54 (3.01)		0.36 (3.05)	
	Late middle-aged adults	–0.33 (3.21)		0.00 (3.11)	
**Educational level**			.67^c^		.08^c^
	Below senior high school	–0.33 (3.53)		–0.42 (3.59)	
	Junior college	0.50 (2.81)		1.31 (2.15)	
	Bachelor and above	0.41 (2.97)		0.06 (2.76)	
**Length of service year**			.39^c^		.36^c^
	<10 years	–0.48 (3.20)		–0.64 (2.87)	
	10-20 years	1.07 (2.97)		0.52 (3.03)	
	>20 years	0.00 (3.01)		0.82 (2.83)	
**Experience of using original information system**			.29^c^		.12^c^
	<3 years	–0.57 (3.33)		–1.05 (3.08)	
	3-6 years	–1.50 (2.38)		0.00 (0.00)	
	>6 years	0.59 (2.99)		0.72 (2.89)	

^a^Difference between original information system and barcode information management system.

^b^Mann-Whitney *U* test.

^c^Kruskal-Wallis test.

**Table 5:** Comparison of participant characteristics and perceived usefulness between nurses and technicians (N=80).

Variables		Perceived usefulness^a^			
		Nurse (n=48) mean (SD)	Technician (n=32) mean (SD)	*P* values	
**Sex**					.08^b^
	Male	0.00 (0.00)	–0.87 (3.37)		
	Female	0.87 (2.48)	–0.41 (3.50)		
**Age (years)**					.08^c^
	Young adults	1.00 (1.67)	0.50 (0.71)		
	Early middle-aged adults	1.04 (2.69)	–1.17 (3.35)		
	Late middle-aged adults	0.47 (2.36)	–0.39 (3.64)		
**Educational level**					.04^c^
	Below senior high school	–0.50 (2.12)	–0.41 (3.73)		
	Junior college	1.71 (2.23)	0.00 (1.22)		
	Bachelor and above	0.45 (2.53)	–2.20 (3.27)		
**Length of service year**					.05^c^
	<10 years	0.85 (1.57)	–1.22 (3.08)		
	10-20 years	0.58 (2.76)	0.37 (3.81)		
	>20 years	1.09 (2.48)	–0.16 (3.97)		
**Experience of using original information system**					.03^c^
	<3 years	0.40 (0.89)	–1.57 (3.43)		
	3-6 years	0.00 (0.00)	0.00 (0.00)		
	>6 years	0.90 (2.58)	0.14 (3.73)		

^a^Difference between original information system and barcode information management system.

^b^Mann-Whitney U test.

^c^Kruskal-Wallis test.

## Discussion

### Principal Findings

This study used an ASD information framework to manage the implementation of a BIMS for operating and supply room staff, focusing on the management of surgical packages. While there was no statistically significant difference in the IMSS scores between the OIS and BIMS, the overall IMSS quality score of the BIMS was higher than that of the OIS. The results demonstrated that 71.2% of the participants had a substantial length of service and over 6 years of experience using the OIS. Previous research has shown that information systems with user-friendly interfaces and ease of operation are advantageous. These benefits should facilitate acceptance by staff in the working environment [26].

This study provided valuable insights into whether nurses perceived system use better than technicians. The findings revealed that nurses indeed had a better perception of system use compared with technicians. Previous research has indicated that Agile transformation is user-based, with attitudes, norms, and self-efficacy being key determinants of intention and healthy behavior [19,27]. These factors are primary concerns in people’s perceptions of Agile transformation. Interventions based on self-determination theory in health settings have been shown to effectively promote the adoption and maintenance of health-related behaviors [28]. Nurses’ better perception of system use compared with technicians can be attributed to their extensive experience, with most nurses in this study having over 20 years of service. In addition, the BIMS is user-friendly, with simplified operations and improved work efficiency. The system’s traceability management of equipment packages also contributed to a stronger sense of identity in clinical care. This enhanced the willingness to use BIMS and encouraged proactive suggestions for system improvements to better meet actual demands.

Regarding perceived usefulness, the subgroup analysis between nurses and technicians showed that educational level and experience using an OIS were statistically significant factors. Effective software development must consider perceived system usefulness to motivate usage [29]. Therefore, the system should align with users’ past experiences, needs, and educational levels to improve outcomes. Operating and supply room nurses are responsible for directing and supervising technicians in performing various sterilization techniques. Nurses involved in developing a surgical BIMS considered standard operating procedures and clinical practice experience. Given that most staff in this study had over 20 years of service and were accustomed to the daily workflow, it was challenging for information engineers to modify the information program immediately during work changes, causing staff pressure. The HIT should improve efficiency and operator satisfaction by aligning workflow and equipment at a reasonable cost [30].

The use of information technology in medical material management significantly reduces staff workload. Acceptance of this technology is influenced by software and hardware availability, user age, and education level. Younger users, with better information skills due to recent education and widespread technology exposure, tend to be more accepting [31,32]. However, insufficient training, concerns about system security, and interdepartmental communication pressures can hinder usage. In addition, changes in workflow, competition for computer access, and extra time required to handle information can reduce interpersonal interactions, further obstructing implementation [32,33]. In the supply room, nurses and technicians collaboratively manage logistics and medical materials. Nurses are responsible for education, guidance, and quality control to ensure the safety of medical materials for patients. BIMS operational functions vary by authority level, with nurses using their system privileges to monitor instrument flow and supply levels, coordinating with clinical processes to enhance recognition and support of clinical care. Literature highlights the importance of defining human-machine collaboration, interactive empowerment, and a digital ecosystem [34]. A strategic management approach to digital quality management, guided by principles of humanistic care, is essential. Continuous development of digital technologies aims to achieve optimal care quality [34,35]. Nursing managers, who also serve as educators, communicators, and supervisors, facilitated the smooth implementation of Agile transition processes through continuous team discussions, workflow adjustments, and ongoing staff education and training.

### Limitations and Recommendations

This study had several limitations. First, a self-reported structured questionnaire was used to measure the effectiveness of the information system. Second, participants were recruited from the operating and supply rooms of a single medical center. Finally, the completion rate was 63%. Based on these limitations, the following recommendations are presented. First, objective instruments should be used to measure the effectiveness of the information system, and qualitative interviews can be conducted to better understand user experience. Second, participants from multiple institutions should be recruited to enhance the external validity of the findings. Third, various strategies should be implemented to increase the completion rates of surveys and questionnaires. Finally, while this study collected quantitative data from nurses and technicians, it is recommended to gather qualitative data in a subsequent phase to validate and complement these findings.

### Conclusions

This study developed a BIMS to replace handwritten information with barcode data. A traceability information management system was established for surgical instruments and packages, facilitating cooperation among the operating room, supply room, software information, and infection control teams to formulate information-based operating specifications. The study re-examined and refined the Agile transformation process through interteam communication. In addition, staff education was emphasized to enhance the acceptability and efficiency of BIMS.

## Abbreviations

ASD: Agile software development
BIMS: barcode information management systems
HIT: health information technology
IMSS: Information Management System Scale
IRB: Institutional Review Board
OIS: original information system
